# Intravascular Biphasic Synovial Sarcoma: The Beneficial Role of Adjuvant Treatment Approach in the Pre-metastatic Stage

**DOI:** 10.7759/cureus.572

**Published:** 2016-04-16

**Authors:** Rodolfo Chicas-Sett, Dolores Farga-Albiol, Erica Collado, Ariel Pacheco, Carlos Zac, Roberto Diaz, Francisco Celada, Javier Burgos, Maria Jose Perez, Alejandro Tormo

**Affiliations:** 1 Radiation Oncology, University Hospital La Fe, Valencia, Spain; 2 Escuela de Doctorado, Universidad Católica de Valencia "San Vicente Mártir"; 3 Radiology, University Hospital La Fe, Valencia, Spain; 4 Pathology, University Hospital La Fe, Valencia, Spain; 5 Oncology, University Hospital La Fe, Valencia, Spain

**Keywords:** synovial sarcoma, intravascular sarcoma, radiotherapy, trabectedin, recurrent thrombosis, biphasic synovial sarcoma

## Abstract

Synovial sarcoma (SS) is a high-grade, rare variant of soft tissue sarcoma (STS). The biphasic subtype is less common than the monophasic subtype. SS is very common around joint cavities in the extremities, but can be present elsewhere in the body. Tumor staging and therapeutic management are usually clear for a localized disease, but the proper management at the metastatic stage can be unclear. According to the literature, the histologic presence of an SS tumor thrombus affects tumor staging, making it unclear whether the tumor stage corresponds to localized or metastatic disease. An intravascular SS tumor exhibiting high metastatic potential is a rare finding that warrants thorough investigation.

A 49-year-old woman presented with a biphasic SS intravascular tumor of the left inguinal area with femoral vessels involvement. Ten cases of intravascular SS have been reported in the literature and contain little information regarding the proper management of a local metastatic disease. Ours is a rare case of SS with an intravascular tumor occupying the femoral-iliac vein (as seen in metastatic disease) that has been treated as a local disease with a multidisciplinary therapeutic approach. As a result, our patient has been disease-free for two years and, during that time, has achieved an acceptable quality of life.

We discuss the pertinent clinical findings of this rare tumor and review the literature of tumor thrombus by SS. We also present the multidisciplinary therapeutic approach realized and the history of this disease.

## Introduction

Soft tissue sarcomas (STS) are rare tumors that develop in supporting tissues. Synovial Sarcoma (SS) accounts for 8 to 10% of all STSs, occurring slightly more often in men than women at a ratio of 1.2:1.0 [1-2]. STS is most often seen in young adults, with a median age of 35 years. These tumors are known to arise in close joint cavities; however, they can occur at any site in the body [2]. Histologically, there are three subtypes of SS: monophasic (spindle cells), biphasic (spindle and epithelial cells), and poorly differentiated. SS includes a characteristic translocation (X; 18) (p11; q11), resulting in the fusion of SYT and SSX1 or SSX2 genes. This translocation can be observed in up to 95% of all SS, making the detection of those fusion genes a diagnostic test for this kind of tumor [3]. Immunohistochemical staining is an integral part of the accurate diagnosis of an SS, thus making it possible to exclude other neoplasms. Between epithelial markers is EMA, CAM 5.2, CK7, and CK19. S-100 is positive in 30% of cases. SMA, desmin, CD34, and CD31 are negative [4].

Treatment for localized SS typically consists of a combination of surgery, radiotherapy (RT) and, in some cases, chemotherapy. Adjuvant therapy in localized SS shows a response rate >65% for chemotherapy and >90% for RT. Nonetheless, the exact benefit of administering systemic therapy once SS is nonlocalized is controversial [5-6].

There are insufficient data at present about epidemiology, diagnosis, and standard treatment in intravascular synovial sarcoma. However, a few intravascular SS have been previously reported.

Herein, we discuss a very rare case of histopathologically confirmed biphasic synovial sarcoma with intravascular tumor thrombus treated with a multidisciplinary therapeutic approach.

## Case presentation

**Figure 1:**
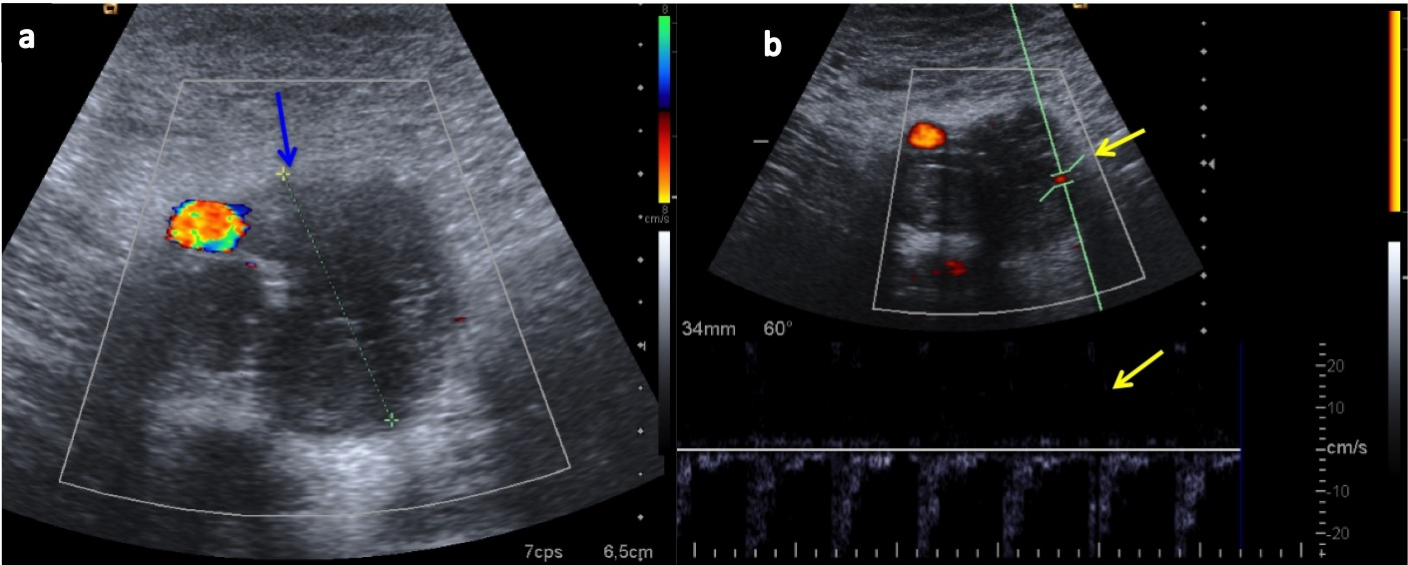
Doppler ultrasound of femoral vein and artery Ultrasound showing a large intraluminal thrombus in the left superficial femoral vein (blue arrow), just adjacent to the femoral artery (a). The pulsed Doppler exploration shows internal vascularization (yellow arrows; b).

**Figure 2:**
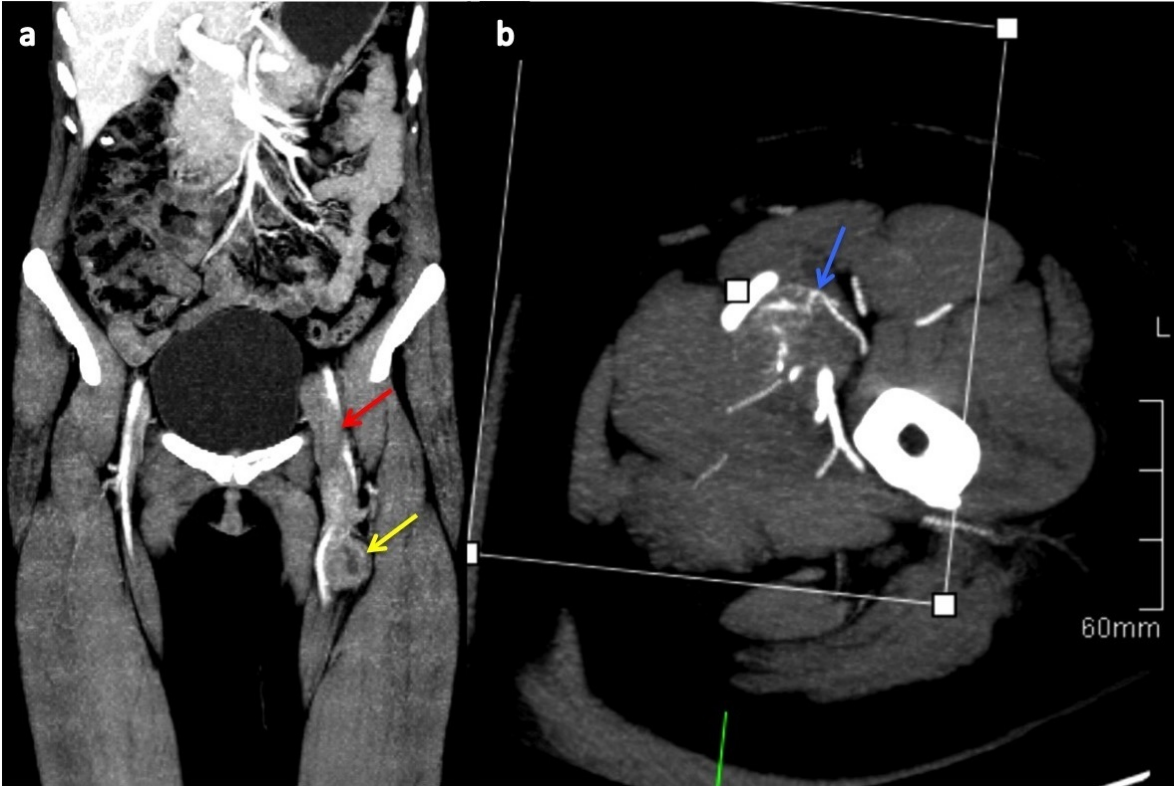
Computed tomography of pelvis and inferior limbs. Venous phase computed tomography with maximum intensity projection (MIP) reconstruction shows a heterogeneous nodular enhancement lesion in the lumen of the left superficial femoral vein (yellow arrow). This caused a venous thrombosis to extend up the common femoral and external iliac veins (red arrow; a). Arterial phase with MIP reconstruction shows an intralesional neovascularization component that suggests tumoral lesion (blue arrow; b).

A 49-year-old woman presented to the emergency unit with a history of swelling and erythema in her left limb after a long car trip. In the absence of a medical history, she was diagnosed with deep vein thrombosis (DVT) via Doppler ultrasound. A month after the diagnosis, she was admitted to an internal medicine service with DVT progression (as noted by ultrasound and computed tomography, presented in Figure 1 and Figure 2), despite appropriate anticoagulant therapy.

**Figure 3:**
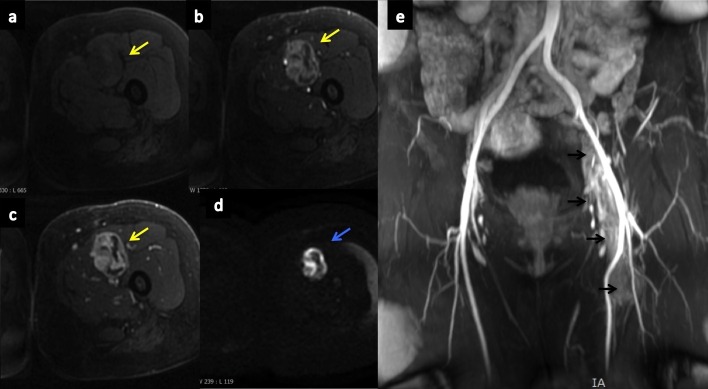
MRI from left femoral vessels Dynamic MRI with gadolinium shows heterogeneous and progressive tumor enhancement (yellow arrows) in the lumen of the left superficial femoral vein (a: T1 FS no contrast, b: T1 FS arterial phase, c: T1 FS venous phase). Diffusion sequence shows restriction (blue arrow) that suggests high cellularity (d). Angio-MRI shows occupation of the lumen of the left superficial femoral vein, common femoral, and external iliac veins by tumoral thrombosis (black arrows; e).

Magnetic resonance imaging (MRI) showed the extension of the thrombosis (external left iliac, common femoral, and the origin of the superficial left femoral) and a nodular form attached to the vessel wall with unclear borders as shown in Figure 3.

A biopsy of the nodular form indicated synovial sarcoma. After histological and immunohistochemical confirmation, the case was presented to the sarcoma committee. No clear evidence of metastatic lesions was observed in the diagnosis images. The multidisciplinary sarcoma board recommended treating the patient according to localized sarcoma protocol, which includes wide surgery excision, RT, and chemotherapy. The insertion of a vena cava filter after surgery was indicated.

Wide surgical excision revealed tumor attachment to the sartorius muscle and tumor infiltration of the femoral vessels (vein and artery). Safe borders were macroscopically accepted. The blood flow was reestablished with a contralateral saphenous bypass. A vena cava filter was placed.

**Figure 4:**
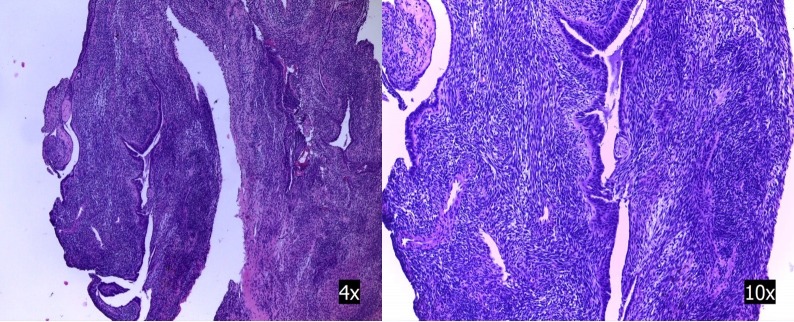
Histologic analysis Atypical biphasic cell proliferation and spindled areas with other epithelial cells.

The pathology was consistent with biphasic synovial sarcoma World Health Organization (WHO) Grade 3. The surgical specimen presented a maximum 9 cm diameter. Tumor embolization, thrombosis, and vascular recanalization were observed. Surgical borders were affected in the proximal area. Figure 4 shows atypical biphasic cell proliferation and spindled areas with epithelial cells.

Immunohistochemistry was positive for CD99, CK1/3, and EMA. SMA, desmin, CD34, pS100, and bcl-2 were negative. Translocation t(X:18) (p11.2;q11.2) was confirmed by FISH.

Ten days after the primary surgery, the patient presented a new episode of thrombosis in the left iliac vein. The thrombus, completely extracted via anterograde and retrograde thrombectomy, was compatible with biphasic synovial sarcoma.

Afterward, adjuvant chemotherapy was administrated with four complete cycles of doxorubicin and ifosfamide.

**Figure 5:**
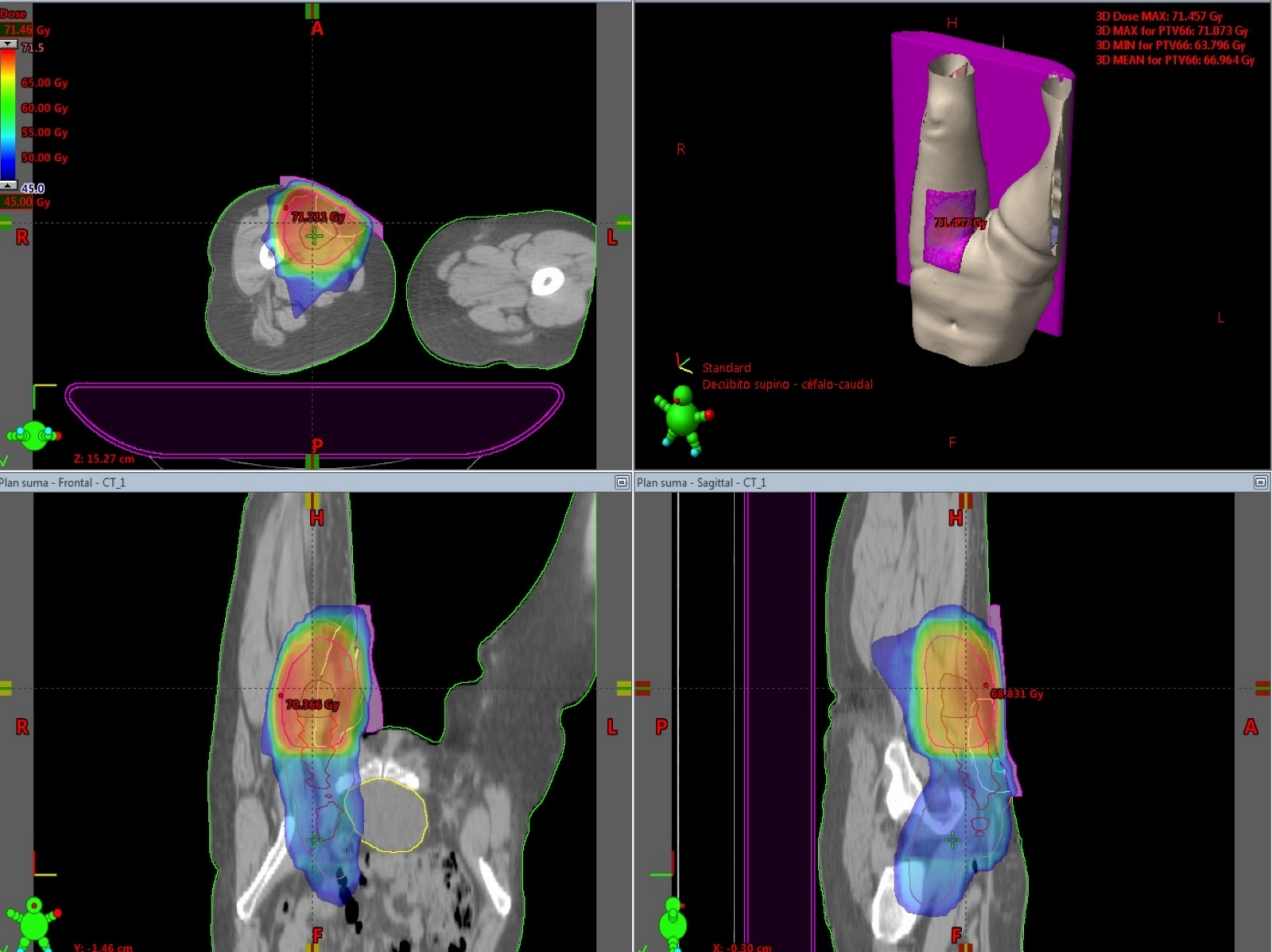
3DRT treatment planning The planned target volume isodose: 66 Gy (surgical bed, scar, and femoral vessels) and 50 Gy (external and internal Iliac, obturator, and inguinal vessels).

The adjuvant RT treatment was performed via 3D-CRT, 6MV Photon beam (Clinac iX VARIAN) through six isocentric and coplanar beams. The treatment dose was prescribed to 95% the planning target volume isodose: 66 Gy (surgical bed, scar, and femoral vessels) and 50 Gy (external and internal Iliac, obturator, and inguinal vessels). The total dose was administrated in 33 fractions (2 Gy per fraction) daily with five fractions per week. Image guide RT was realized through Kv/Kv and Cone Beam. Figure 5 shows the radiotherapy planning treatment administrated to the patient.

The patient was disease-free for two years with strict medical visits and a very good quality of life. 

A new episode of tumor thrombosis in the femoral vein developed. We performed a thrombectomy and noted a synovial sarcoma thrombus with the same immunohistochemical characteristics.

Given the patient’s exceptional case, multiple recurrence risk factors, and the controversy around the use of adjuvant therapy for local metastatic disease, the sarcoma board recommended eight cycles of trabectedin for the patient.

As of this writing, the patient is free of disease and undergoes frequent medical follow-ups. The treatment was well-tolerated. No acute and late toxicities were reported.

## Discussion

After a comprehensive review of the literature, we have determined that intravascular manifestations of sarcomas are extremely rare. Schreiner et al., reported only ten documented cases of intravascular synovial sarcoma [7]. Schreiner et al., noted a higher incidence in women, and patient age ranges from 16 to 54 years old, with the predominant age group appearing to be 30 to 40 years old. The biphasic tumor subtype was more prevalent. Pain and swelling in a limb were the most common symptoms. The proximal femoral and iliac veins were the vessels most often affected. Schreiner et al reported two cases with involvement of the inferior vena cava. Individual cases of pulmonary emboli were also described [7].

In our case, it was very difficult to establish if the origin of the primary tumor was within the vessel or outside of the vessel. However, the radiological studies and pathological results were consistent with those expected in a rare case of intravascular sarcoma. One of the first areas of uncertainty, in this case, was the staging. The intravascular sarcoma (tumor thrombus) is not contained in the TNM staging system, but we believe it correlates to a 'pre-metastatic' disease due to its high metastatic potential.

The treatment options available for SS are wide surgical removal of the lesion and RT, often in combination with chemotherapy [8].

In this case, immunohistochemical determination of biphasic synovial sarcoma was established in the primary lesion (the intravascular tumor and mass attached to the vessel) localized in a femoral vessel. A new thrombosis episode presented ten days following primary surgery, localized in the iliac vein. The thrombus was removed, and the pathological analysis revealed the same histology and epithelial markers. Indeed, this new thrombosis was concerning because it may indicate the persistent presence of the microscopic primary tumor. Given the disease’s characteristics, RT to the primary site (femoral vessel) and iliac vein was included in the treatment planning, and adjuvant chemotherapy (doxorubicin-ifosfamide) was administrated. 

A local metastatic treatment (i.e., surgery, RT) may alleviate symptoms, prolong progression-free survival, or cure selected patients, depending on the metastatic burden. These treatments are associated with chemotherapy delivered sequentially, perioperatively, or as exclusive therapy. Kavanagh et al., note two premises justifying the therapeutic strategy for metastatic burden sarcomas: clinical experience suggests that selected patients benefit from metastatic ablation, and consolidative local therapy produces good results in selected partial responders to systemic therapy [9-10].

Trabectedin, approved in Europe in 2007 for the treatment of advanced metastatic soft-tissue sarcomas, has shown beneficial effects after failure with standard chemotherapy. The evidence suggests that translocation-related sarcomas may be sensitive to trabectedin [10].

Considering the extreme rarity of intravascular synovial sarcomas, the feasibility of adjuvant treatment in a metastatic burden stage using local interventions (surgery, RT) and the use of new drugs must be evaluated to determine the future clinical indications in similar cases. In our case, the patient was disease-free for two years after primary treatment. Following a relapse, salvage surgery and trabectedin were used. At present, the patient has no evidence of the disease.

## Conclusions

Intravascular synovial sarcomas are extremely rare lesions, and the most effective treatment is unknown. There are ten similar cases reported, but this case is the first to use local interventions and trabectedin. As the efficacy of adjuvant treatment for 'pre-metastatic' intravascular SS has not been widely studied because the disease is rare, our case indicates traditional treatments and new systemic drugs can improve the quality of life and delay the metastatic stage.
